# Minimally Invasive Guidewire-Aided Bladder Diverticulectomy with or Without Other Prostatic Intervention: Step by Step Description of the Procedure

**DOI:** 10.3390/jcm14061899

**Published:** 2025-03-12

**Authors:** Bernardo Rocco, Alessandro Antonelli, Maria Chiara Sighinolfi, Enrico De Marzo, Simone Assumma, Luca Sarchi, Enrico Panio, Tommaso Calcagnile, Giorgio Bozzini, Riccardo Bertolo, Marinella Finocchiaro, Hakan Görkem Kazıcı, Aryan Pathak, Marcio Covas Moschovas, Vipul Patel, Mario Falsaperla

**Affiliations:** 1 Unit of Urology, ASST Santi Paolo and Carlo, University of Milan, 20122 Milan, Italy; bernardo.rocco@gmail.com (B.R.); simone.assumma@gmail.com (S.A.); luca.sarchi88@gmail.com (L.S.); enrico.panio@gmail.com (E.P.); tommasocalcagnile93@gmail.com (T.C.); 2 Department of Urology, University of Verona, 37129 Verona, Italy; alessandro.antonelli@univr.it (A.A.); riccardobertolo@hotmail.it (R.B.); 3 Department of Urology, Regional Health Care and Social Agency Civil Hospitals of Brescia, 25123 Brescia, Italy; enrico.demarzo89@gmail.com; 4 Department of Urology, ASST Lariana, 22100 Como, Italy; gioboz@yahoo.it; 5 Department of Urology, ARNAS Garibaldi Hospital, 95123 Catania, Italy; marinellafinocchiaro@gmail.com (M.F.); mayurol@yahoo.it (M.F.); 6 Aydın State Hospital Urology Clinic, Aydın 09100, Turkey; hgkazici@yahoo.com; 7 Department of Urology, AdventHealth Global Robotics Institute, Kissimmee, FL 34747, USA; aryanpathak@ufl.edu (A.P.); marcio.doc@hotmail.com (M.C.M.); vipul.patel.md@adventhealth.com (V.P.)

**Keywords:** robotic surgery, laparoscopy, bladder, diverticulectomy, new technique

## Abstract

**Introduction**: Laparoscopic and robotic bladder diverticulectomy is a successful option to correct bladder diverticula (BD). Nevertheless, the identification of BD could be a tricky step, due to the presence of pneumoperitoneum compressing the bladder. This occurrence could be particularly evident for the posterior or postero-lateral location of BDs. We present a novel technique to overcome this concern based on a rigid guidewire previously endoscopically placed and coiled inside BD, to ensure it expands and remains stable during the dissection. The technique was used in cases of diverticulectomy concomitant to other prostatic procedures. **Methods**: This is a multicentric series of laparoscopic and robotic diverticulectomy performed with this original technique in 34 patients. The procedure was concomitant to other prostatic intervention in most of the cases: TURP or bladder neck incision (16); radical prostatectomy (three); Millin adenomectomy (four cases). Surgical procedure: The first step of the procedure endoscopic, consisting of the retrograde insertion of a stiff guidewire inside the BD via cystoscopy; the guidewire is pushed in until it coils inside the diverticulum, and then enlarged to make it visible transperitoneally. The guidewire stretches the diverticulum and guides the dissection up to identify its neck. The primary endpoint is to address the feasibility of the technique by considering the operative time (OT, min) and the complication rate. **Results**: The median size of the BDs was 5.1 cm. The location of the BD was postero-lateral or posterior in all except one case. Bladder diverticulectomy was laparoscopically performed in 25 and robotically assisted in nine cases. Median OT was 179 min (DS 42). The post-operative course was uneventful for all except two patients with symptomatic urinary tract infections. **Conclusions**: The use of a stiff guidewire coiling and expanding the BD is a simple and useful trick to aid BD’s identification and dissection; it aids diverticulectomy and is also concomitant to other prostatic procedures.

## 1. Introduction

Bladder diverticula (BD) are abnormal distension of the bladder wall, typically resulting from increased intravesical pressure due to bladder outlet obstruction (BOO). The pathogenesis of acquired bladder diverticula is closely associated with conditions that cause chronic obstruction, such as benign prostatic hyperplasia (BPH), bladder neck obstruction, or neurogenic bladder dysfunction. When the bladder experiences sustained elevated pressure from impaired voiding, the weakened areas of the bladder wall, often at the junction of the detrusor muscle and mucosa, herniate outward, forming diverticula [1]. Histologically, the diverticulum consists of mucosa and submucosa, but it lacks a full muscular layer, which can contribute to its susceptibility to infection and dysfunction [2]. In the context of BOO, the detrusor muscle becomes hypertrophic and less efficient, while the bladder capacity may decrease, leading to progressive symptoms of lower urinary tract dysfunction. The persistent increase in intravesical pressure, combined with the detrusor muscle’s inability to fully evacuate the bladder, facilitates the formation and enlargement of diverticula. In some cases, congenital factors may predispose to bladder diverticula, but acquired diverticula are far more common in the adult population. Treatment typically involves addressing the underlying obstruction and may include surgical interventions like transurethral resection or diverticulectomy [3].

Initially defined as open surgery and performed in this manner for many years, bladder diverticulectomy began to be performed with minimally invasive techniques with the development of technology. Transurethral fulguration of bladder diverticulum, first defined in the 1970s, was used for many years as an alternative to open surgery in various patients [4]. Laparoscopic diverticulectomy was defined in the early 1990s as laparoscopic techniques began to be used in urological diseases [5] and this method has been gradually developed over time and has been successfully applied by urologists for more than 30 years. With the advent of the 2000s and the development of robotic surgery, the first robotic diverticulectomy was defined by Berger and Stifelman in 2006 [6]. Following these definitions, innovations were also made to this robotic method by different surgeons; thus, techniques were gradually improved and alternative surgeries were created.

During the last decade, laparoscopic and robotic diverticulectomy has strengthened its place as a successful option to correct bladder diverticula as the gold standard. The onset of symptoms—i.e., urinary tract infection and/or stones—represents a clear indication to diverticulectomy. To note, if left uncorrected, ab-extrinseco obstruction of ureter or bladder cancer could be further drawbacks and surgical management are strongly recommended to avoid the evolution toward these harmful conditions too [7,8,9,10,11].

Laparoscopic and robotic surgery addresses the need for a minimally invasive management of this benign condition; furthermore, it enables the concomitant treatment of the underlying condition causing the diverticulum. The downside of pure and robotic laparoscopy is the pneumoperitoneum that compresses the bladder and makes the diverticulum less recognizable under the intact peritoneum, making bulge identification the most challenging part of the procedure [7,8,9]. Several techniques were described to overcome this problem, but most of them add operative time and complexity without being definitively beneficial.

Guidewires are surgical equipment that have been used safely in many urological procedures for many years. They are used especially in kidney and ureter surgeries to place double J stents and to provide a straight structure for the ureter [12]. On the other hand, we know that they are used in the insertion of the catheter without damaging the urethra in urethral strictures [13]. Guidewires can sometimes be used to change the position and posture of urinary organs. For example, in a newly developed technique, it was stated that they used guidewires to elevate the roof of the ureter during injections into the ureter in VUR patients in order to increase the comfort of the surgical technique [14]. We also developed a new surgical technique using guidewires in a technique that has never been used before to determine the location and boundaries of bladder diverticula during robotic and laparoscopic surgery.

The aim of the article is to describe a novel method to identify the diverticulum and to facilitate its isolation, enabling an easy and time-sparing procedure. The technique consists of a rigid guidewire cystoscopically placed and coiled inside the diverticulum for it to be expanded and stable during the dissection. We present a multicentric series of laparoscopic and robotic diverticulectomy performed with this approach, concomitant to other prostatic procedures in most of the cases.

## 2. Methods

This is a retrospective analysis of a prospectively collected series of patients undergoing laparoscopic and robotic diverticulectomy in three urological departments from Italy (Chiefs: M.F; B.R; A.A.). The initial work-up included medical history, physical examination, pre-operative cystogram, abdomen ultrasound or contrast CT scan, and urine culture. Baseline characteristics were recorded, including age, size of the BD (maximum diameter), location of the BD, operative time (minutes), intra- and post-operative complications, time to catheter removal, and length of hospital stay. Follow up was assessed at 3 weeks, 6 months, and 12 months from surgery, by means of clinical examination, blood and urine tests, and abdominal ultrasonography.

All patients signed an informed consent to the procedure that included the description of surgical steps; the current should be considered a simple variation in an established procedure (diverticulum distension), rather than a novel technique.

## 3. Surgical Technique

Cystoscopy: The first endoscopical step was performed to rule out bladder cancer, to identify BD, and eventually to insert ureteral stents mono- or bilaterally, to promptly identify the pelvic ureter and its relationship with the BD during dissection and the ureteral orifice during the bladder suture.

**Figure 1:**
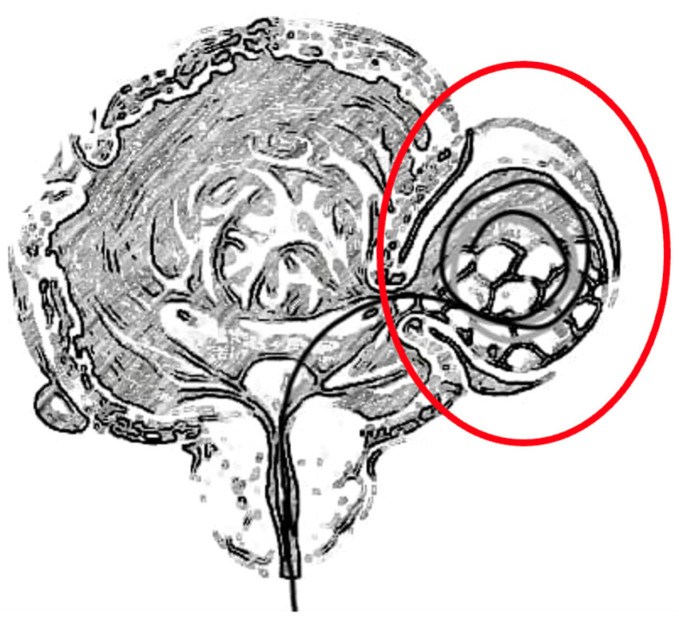
The guidewire is inserted with cystoscopy through the ostium of the diverticulum (red circle) and is coiled inside several times.

Afterwards, a stiff guidewire (Terumo or Sensor) is placed inside each diverticulum and pushed in, until it coils inside several times (Figure 1). The characteristics of the guidewire should be resistance, as provided by a nitinol core, hydrophilic coating to allow for lubricity, and a gentle tip for smooth and rapid movement through difficult tissues to avoid the disruption of the wall of the diverticulum. The placement occurs under vision or under fluoroscopic assistance. The guidewire aids the identification of BD through the peritoneum, since it allows the operator to recognize “at a glance” the diverticulum filled and stretched, keeping the urinary bladder empty. In addition, during the next dissection, the guidewire easily allows the operator to reach the BD neck. After that, a urethral catheter was inserted in the bladder to ensure it remains empty. Then, the guidewire is fixed to the transurethral catheter to avoid its displacement when moving to the anterior lap/robotic abdominal accesses. During the change in patient positioning (from lithotomic to supine position, i.e., with the robotic DV Xi approach), the guidewire could be accidentally displaced: particular attention should be paid at this point, with a dedicated nurse/personnel taking care of the catheter and the guidewire while holding them.

**Figure 2:**
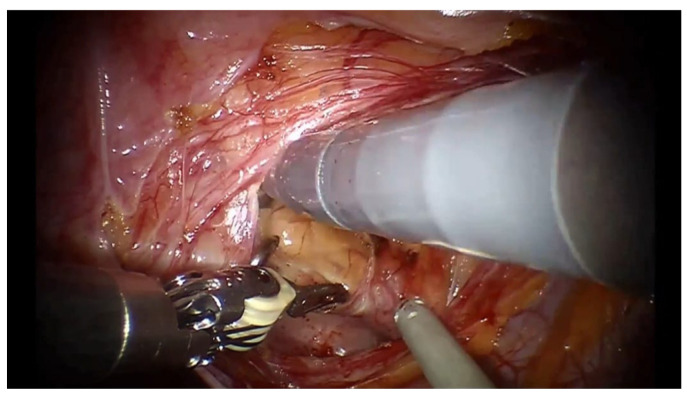
The peritoneum on the diverticulum is incised.

**Figure 3:**
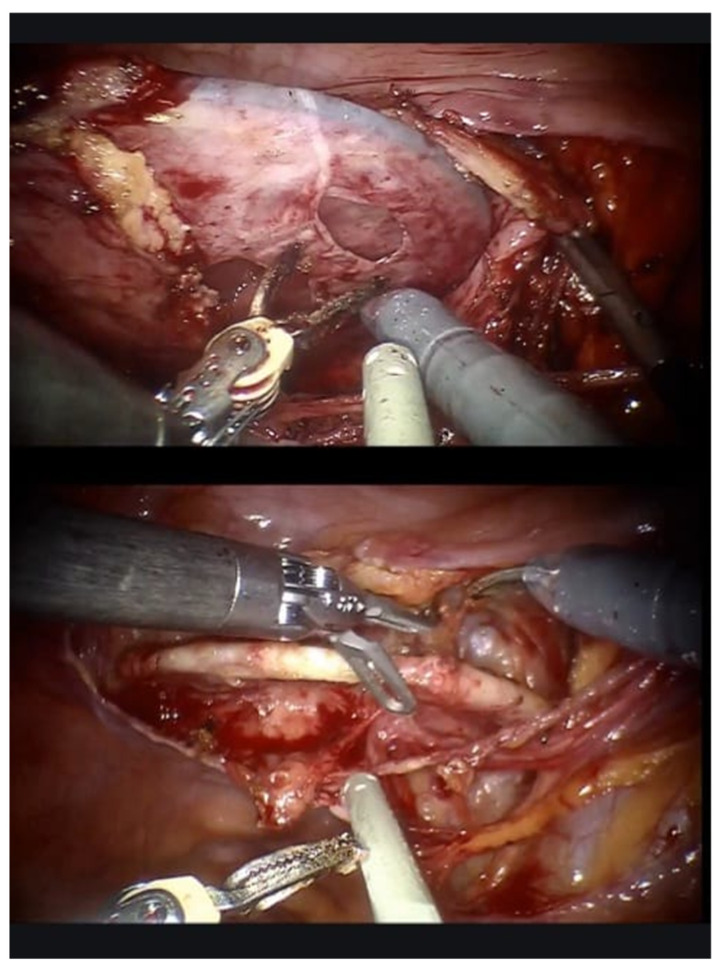
As the dissection of the diverticulum proceeds, the diverticulum gains an elipsoid aspect due to the underneath guidewire that keeps it stretched. At this point, the bladder diverticulum can be incised, and the guidewire is accessible transperitoneally.

Laparoscopic/robotic diverticulectomy: trocar placement was performed as usually carried out for pelvic surgery. The ureter homolateral to BD was identified. BD is visible as a rigid and expanded bulge in the peritoneum, stretched by the previously coiled guidewire; additionally, the external manipulation of the guidewire can further facilitate BD by its movement. The peritoneum over the BD is incised (Figure 2) to realize a plane between it and the peri-vesical fat; the bladder and BD are grasped and swept free of their peritoneal attachments. The BD is then isolated with sharp and blunt dissection, with low monopolar cautery use. As isolation proceeds, the BD gains a discoidal appearance (Figure 3); the firm coils of the guidewire, clearly visible through the BD wall, could be easily grasped to aid in finding the correct plane of dissection. The dissection should be performed circumferentially for complete BD mobilization and proceed up to the diverticular neck. Care should be taken not to enter the BD at the beginning; in such a case, the inner vision of the BD with the precise identification of the BD neck thanks to the guidewire eventually allows the operator to adapt the resection strategy. The BD neck can be first circumferentially excised and then the BD mucosa dissected. Once completely isolated toward its ostium, the BD is opened and dissected. The bladder defect is closed with a double layer 3-0 monocryl running suture. A video of the procedure is available at the following link: https://www.youtube.com/watch?v=SI6PnKGiM1U (accessed on 25 November 2024).

Associated procedure to treat bladder outlet obstruction: Endoscopic procedures (TURP, TUIP, BNI) were performed as the first step, prior to the procedure of resection of the BD; at the end, after accurate haemostasis, a standard 3 ways catheter was placed. Adenomectomy was instead performed after BD excision and bladder closure; the Retzius space was entered through a supravescical peritoneum incision and minimally invasive Millin’s adenomectomy was performed. Robotic radical prostatectomy was carried out after robotic diverticulectomy.

## 4. Results

A total of 34 patients were collected among three urological departments performing laparoscopic and robotic urological surgery. The median age of the patients was 64.9 years (DS 10.7). Moreover, 29 out of 34 patients had a singular diverticulum, whereas 5 patients had at least two diverticula. The median size of the BDs was 5.1 cm (DS 1.21, range 3–8 cm). The location of the BD was postero-lateral in 20 cases, posterior in 13, anterior in one case. Bladder diverticulectomy was carried out laparoscopically in 25 patients and was robotically assisted in the remaining nine patients. The procedure was performed concomitant to TURP or bladder neck incision (16); radical prostatectomy (three); and Millin adenomectomy (four cases). The median operative time was 179 min (DS 42, range: 120–300 min). The median time to catheter removal was 7.9 days (DS 1.8, range 5–13). The median length of hospital stay was 6.5 days (DS 1.66, range 2–12). Only 8/34 patients had a post-operative cystogram, as clinically indicated; none of them had urinary leakage 6–11 days after surgery. The post-operative course was uneventful for all except two cases who had post-operative fever and urinary tract infection. Table 1 summarizes the pre-, intra-, and post-operative variables. As a note on technique, particular care should be taken not to displace the guidewire while changing patient positioning and moving to the abdominal field. However, the technique was accomplished successfully in all cases and no differences related to the various concomitant interventions were noticed.

Twelve-months follow up was available in more than half of the cases; blood and urine tests and abdomen ultrasound were negative for anomalous bladder findings and/or UTI at all steps of the follow up.

## 5. Discussion

Laparoscopic and robotic bladder diverticulectomy is a feasible procedure that could be performed concomitant to the surgical treatment of the underlying condition causing the BD (either endoscopical, pure laparoscopical, or robotically assisted) [7,8,9]. The technique we reported in the current paper is a simple way to overcome one of the most concerning steps of the laparoscopic and robotic approaches, namely the identification of the BD. Diverticula could hardly be identified especially if they are posterior and postero-laterally located or in the case of postprocedural edema and imbibition of tissues. From our series, the use of a rigid guidewire—previously endoscopically positioned—keeps the dome of the diverticulum expanded and identifiable; despite its smooth and mobile wall, the guidewire fixes and limits the movement of the diverticulum during dissection and manipulation. Advantages are a clear view of the whole surface of the diverticula and less of a chance to inadvertently enter it. Furthermore, the firmness provided by the stiff guidewire limits the need for continuous countertraction to hold back redundant diverticular tissue. In our series, this trick enabled an easy identification of the BD through the peritoneum and a simple dissection, resulting in acceptable operative times without added morbidity. The concern of the identification of a BD through a laparoscopic and robotic approach was addressed by previous authors with different techniques.

At the very beginning of the establishment of the procedures, some authors described the use of cystoscopic transillumination, performed intra-operatively by the bedside assistant, by turning off robotic lights, which may help identify the location and extension of the diverticulum [8]. Similarly, the use of endolaparoscopic ultrasound was proposed by Porpiglia et al. [15] to enhance the visualization of the diverticulum through the peritoneum, with a safe and non-invasive approach. Other authors suggested tools to make the diverticulum more identifiable and expanded. A first approach consisted of the insertion of a urethral catheter inside the diverticulum, with the balloon inflated with 20–30 mL: the traction of the balloon occluding the diverticular neck allows for the filling of the diverticulum and its easy identification [16,17]. A drawback of this technique is the frequent dislodgement of the catheter from a wide neck, making such an effort ineffective. Furthermore, urethra may not be compliant to accommodate two urethral catheters. To facilitate diverticulum identification, Flasko et al. [18] made up a thinner diverticular catheter made of a 7F ureteral catheter covered by a piece of surgical glove. Injection of fluid into the catheter would inflate the glove and allow for the identification of the diverticulum. Finally, Tareen et al. [19] proposed an endoscopic resection of bladder diverticulum concomitant to the laparoscopic observation from an intraperitoneal view to avoid damage to surrounding tissues. Noticeably, this approach could not fit robotic surgery, since it requires undocking and represents only an intraperitoneal control of an endoscopic management of diverticulum. More recently, the use of the Firefly Fluorescence Imaging Da Vince System (FFIS) was reported on four cases of robotic bladder diverticulectomy to enhance the transillumination effect and make the diverticulum more identifiable intra-operatively [20].

Giannarini et al. [21] reported on the use of a transperitoneal robotic approach on 16 patients: in their series, BD were identified through bladder distension with saline through an indwelling catheter with or without concomitant illumination using flexible cystoscopy and fluorescence imaging. Another surgical technique was published by Develtere et al. [22] who used a robotic transperitoneal approach on 23 patients. However, unlike previous authors, they described the resection of the diverticulum via cystotomy by performing an incision of the mucosa covering the diverticular neck and by identifying the plane between the mucosa and the muscularis. The mucosa is then separated from the surrounding structures and the base of the diverticulum is transected and the defect closed with a barbed suture. A transvesical management of BD was also described in other series [23,24]; the use of the Da Vinci single port was reported in some cases [25,26], in which an extra peritoneal and transvesical approach can be pursued as well.

Hattori et al. [27] described a technique in which diverticulectomy is performed using robotic arms through the bladder neck that is opened during prostatectomy. Since the procedure is performed through an incision in the bladder neck, it is anatomically more suitable for the treatment of diverticula in the posterior wall, but it is an exploratory technique that has only been tried on three patients. Bologna et al. [28] performed a case with LUTS and bladder diverticulum by combining the Firefly-guided technique with single port robotic simple prostatectomy. The authors stated that this technique can be applied transvesically or transperitoneally, but the transperitoneal approach reduces the possibility of intra-abdominal injury, especially for cases in the upper wall.

Kajaia et al. [29] performed robotic surgery on 13 patients with bladder diverticulum and performed other surgeries simultaneously in these cases, as we did in our series. They stated that simultaneous surgeries such as YV bladder neck plasty, prostate adenoma enucleation, and inguinal herniotomy could be performed successfully and did not detect any measurable complications according to the Clavien–Dindo classification. Similarly, we performed simultaneous additional prostate surgery in 23 of the cases included in our study and did not experience any significant complications in these cases.

Gibson et al. [30] performed robotic diverticulectomy using intravesical and extravesical surgical techniques on 26 patients with benign and malignant diverticula. Good functional results were achieved in the diverticula that developed due to benign disease. The success rate was seen to be around 90% in the diverticula that contained malignancy. Although the authors stated that they performed the surgery with attention to oncological principles, case series for malignancy indication are limited and more studies are needed to evaluate the safety of this technique.

Ge et al. [31] performed robotic diverticulectomy on three patients with papillary tumor lesions in bladder diverticula. In this method, the authors completed the diverticulectomy under robotic vision by filling the bladder with distilled water to expand the diverticulum and identify the borders of the diverticulum. The authors argued that the distilled water they used not only helped to visualize the borders of the diverticulum but also reduced free tumor cells in the bladder. They recommended draining the distilled water from the bladder before incision was made after the diverticulum was identified. The authors stated that no local recurrence or distant metastasis developed in any patient during approximately 2 years of follow up. However, it should be noted that the use of this technique in only three cases is insufficient to make a general comment.

Sun et al. [32] performed robotic diverticulectomy using the da Vinci Xi in a patient with a 13 cm giant bladder diverticulum. The authors demonstrated that robotic surgery can be used even in giant diverticula because the robotic system provides a clearer view of the anatomical connections among the ureter, prostate, and diverticulum. This is valuable because it shows that it can be applied safely in more extreme cases. However, they did not mention the technique they used to define the diverticula in the article. Koehne et al. [33] successfully performed robotic diverticulectomy and simultaneous extravesical ureteral reimplantation in a 7-year-old patient with congenital paraureteral bladder diverticulum. Considering that bladder diverticula are not always the result of acquired bladder outlet obstruction and that some diverticula are congenital, it is important that robotic diverticulectomy can be successfully performed in children because of less pain and faster recovery.

To our knowledge, we report the first transperitoneal extravescical management with a guidewire-assisted identification of the diverticulum, especially with regard to performed concomitant on other prostatic procedures in most of the cases. This simple strategy provides advantages also in terms of dissection, given the firmness and fixation of BD walls provided by the stiff and coiled guidewire.

By making the whole procedure easier, this trick may reduce operative time as well. Another strength is the simple, fast, and routine feature of the added procedure—cystoscopy and guidewire positioning—which is safe and cost-effective with limited added operative time.

Even if not recorded within the structured questionnaires, the technique looked simple according to all surgeons with immediate reproducibility. Provided endourology is a part of urologists’ skills armamentarium, the retrograde approach to the bladder and to the diverticula did not represent a barrier to the procedure.

The main limitation of the article is the sample size, even though it is larger than some reported in the literature. The absence of details about the operative time required for the endourological step is another one. No comparison with other techniques to expand the diverticulum is achieved within this series; this could be seen as another limitation. Furthermore, another limit could be the development and use of the technique in referral centers from laparoscopic and robotic surgeons who have already completed their learning curve; its use from novice surgeons (i.e., residents) has not been tested yet. Recognizing the issue, the strength of the current study is its multicenter fashion with the technique being proved and immediately applied in different centers, thus confirming its simple reproducibility across different series and laparoscopic and robotic approaches.

From a first meeting report on the technique [34], the enrollment and indication to BD alone within minimally invasive series is still going on and further generalizability of the technique should be tested on a wider sample size.

## 6. Conclusions

Laparoscopic and robotic bladder diverticulectomy with this original technique may represent a simple and seemingly reproducible approach for the correction of BD, including in cases of concomitant to the treatment of prostate disease or bladder outlet dysfunction. Further implementation of the technique by surgeons of different backgrounds (i.e., novices) is ongoing and results will be soon available.

## Figures and Tables

**Table 1:** Reports of the pre-, intra-, and post-operative characteristics of the cohort.

Pre-Operative Characteristics	
Age	64.9 (DS 10.7)
Size of the diverticulum	5.1 cm (DS 1.2)
Singular diverticulum	29 (85%)
Multiple diverticula	5 (15%)
Location	
Postero-lateral	20 (58%)
Posterior	13 (34%)
Anterior	1 (3%)
Approach	
Laparoscopic	25 (73%)
Robotic	9 (27%)
Concomitant procedures	
TURP or bladder neck incision	16
Radical prostatectomy	3
Millin adenomectomy	4
**Intra- and Post-Operative Characteristics**	
Operative time	179 min (DS 42)
Time to catheter removal	7.9 days (DS 1.8)
Length of hospital stay	6.5 days (DS1.6)
Complication rate	
Clavien II	2 (5.8%)
Clavien III or more	0

## Data Availability

The original contributions presented in the study are included in the article, further inquiries can be directed to the corresponding author.
